# Piecewise Approximate Bayesian Computation: fast inference for discretely observed Markov models using a factorised posterior distribution

**DOI:** 10.1007/s11222-013-9432-2

**Published:** 2013-11-29

**Authors:** S. R. White, T. Kypraios, S. P. Preston

**Affiliations:** 1 MRC Biostatistics Unit, Cambridge, CB2 0SR UK; 2 School of Mathematical Sciences, University of Nottingham, Nottingham, NG7 2RD UK

**Keywords:** Approximate Bayesian Computation, Simulation, Stochastic Lotka–Volterra

## Abstract

Many modern statistical applications involve inference for complicated stochastic models for which the likelihood function is difficult or even impossible to calculate, and hence conventional likelihood-based inferential techniques cannot be used. In such settings, Bayesian inference can be performed using Approximate Bayesian Computation (ABC). However, in spite of many recent developments to ABC methodology, in many applications the computational cost of ABC necessitates the choice of summary statistics and tolerances that can potentially severely bias the estimate of the posterior.

We propose a new “piecewise” ABC approach suitable for discretely observed Markov models that involves writing the posterior density of the parameters as a product of factors, each a function of only a subset of the data, and then using ABC within each factor. The approach has the advantage of side-stepping the need to choose a summary statistic and it enables a stringent tolerance to be set, making the posterior “less approximate”. We investigate two methods for estimating the posterior density based on ABC samples for each of the factors: the first is to use a Gaussian approximation for each factor, and the second is to use a kernel density estimate. Both methods have their merits. The Gaussian approximation is simple, fast, and probably adequate for many applications. On the other hand, using instead a kernel density estimate has the benefit of consistently estimating the true piecewise ABC posterior as the number of ABC samples tends to infinity. We illustrate the piecewise ABC approach with four examples; in each case, the approach offers fast and accurate inference.

## Introduction

Stochastic models are commonly used to model processes in the physical sciences (Wilkinson 2011a; Van Kampen 2007). For many such models the likelihood is difficult or costly to compute making it infeasible to use conventional inference techniques such as maximum likelihood estimation. However, provided it is possible to simulate from a model, then “implicit” methods such as Approximate Bayesian Computation (ABC) methods enable inference without having to calculate the likelihood. These methods were originally developed for applications in population genetics (Pritchard et al. 1999) and human demographics (Beaumont et al. 2002), but are now being used in a wide range of fields including epidemiology (McKinley et al. 2009), evolution of species (Toni et al. 2009), finance (Dean et al. 2011), and evolution of pathogens (Gabriel et al. 2010), to name a few.

Intuitively, ABC methods involve simulating data from the model using various parameter values and making inference based on which parameter values produced realisations that are “close” to the observed data. Let the data *x*=(*x*_1_,…,*x*_*n*_)≡(*x*(*t*_1_),…,*x*(*t*_*n*_)) be a vector comprising observations of a possibly vector state variable *X*(*t*) at time points *t*_1_,…,*t*_*n*_. We assume that the data arise from a Markov stochastic model (which encompasses IID data as a special case) parameterised by the vector *θ*, which is the target of inference, and we denote by *π*(*x*∣*θ*) the probability density of the data given a specific value of *θ*. Prior beliefs about *θ* are expressed via a density denoted *π*(*θ*). Algorithm 1 generates *exact* samples from the Bayesian posterior density *π*(*θ*∣*x*) which is proportional to *π*(*x*∣*θ*)*π*(*θ*).

**Algorithm 1:**
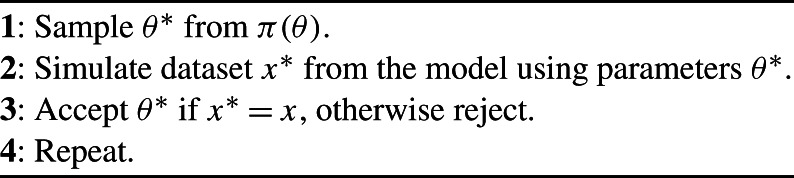
Exact Bayesian Computation (EBC)

This algorithm is only of practical use if *X*(*t*) is discrete, else the acceptance probability in Step 3 is zero. For continuous distributions, or discrete ones in which the acceptance probability in step 3 is unacceptably low, Pritchard et al. (1999) suggested Algorithm 2, where *d*(⋅,⋅) is a distance function, usually taken to be the *L* ^2^-norm of the difference between its arguments; *s*(⋅) is a function of the data; and *ε* is a tolerance. Note that *s*(⋅) can be the identity function but in practice, to give a tolerable acceptance rate, it is usually taken to be a lower-dimensional vector comprising summary statistics that characterise key aspects of the data.

**Algorithm 2:**
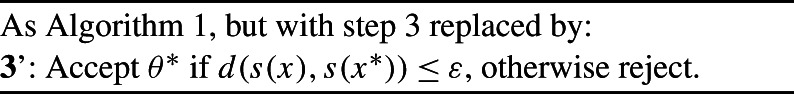
Approximate Bayesian Computation (ABC)

The output of the ABC algorithm is a sample from the ABC posterior density $\tilde{\pi}(\theta\mid x) = \pi(\theta\mid d (s(x), s(x^{*}))\leq\varepsilon)$. Provided *s*(⋅) is sufficient for *θ*, then the ABC posterior density converges to *π*(*θ*∣*x*) as *ε*→0 (Marin et al. 2012). However, in practice it is rarely possible to use an *s*(⋅) which is sufficient, or to take *ε* especially small (or zero). Hence ABC requires a careful choice of *s*(⋅) and *ε* to make the acceptance rate tolerably large, at the same time as trying not to make the ABC posterior too different from the true posterior, *π*(*θ*∣*x*). In other words, there is a balance which involves trading off Monte Carlo error with “ABC error” owing to the choice of *s*(⋅) and tolerance *ε*.

Over the last decade, a wide range of extensions to the original ABC algorithm have been developed, including Markov Chain Monte Carlo (MCMC) (Marjoram et al. 2003) and sequential (Toni et al. 2009; Dean and Singh 2011) implementations, the incorporation of auxiliary regression models (Beaumont et al. 2002; Blum and François 2010), and (semi-)automatic choice of summary statistics (Fearnhead and Prangle 2012); see Marin et al. (2012) for a review. In all of these ABC variants computational cost is still a central issue, since it is always the computational cost that determines the balance that can be made between controlling Monte Carlo error and controlling bias arising from using summary statistics and/or non-zero tolerance.

In this paper we propose a novel algorithm called *piecewise ABC* (PW-ABC), the aim of which is to substantially reduce the computational cost of ABC. The algorithm is applicable to a particular (but fairly broad) class of models, namely those with the Markov property and for which the state variable is observable at discrete time points. The algorithm is based on a factorisation of the posterior density such that each factor corresponds to only a subset of the data. The idea is to apply Algorithm 2 for each factor (a task which is computationally very cheap), to compute the density estimates for each factor, and then to estimate the full posterior density as the product of these factors. Taking advantage of the factorisation lowers the computational burden of ABC such that the choice of summary statistic and tolerance—and the accompanying biases—can potentially be avoided completely.

In the following section we describe PW-ABC in more detail. The main practical issue of the method is how to use the ABC samples from each posterior factor to estimate the full posterior density. We discuss two approaches to estimating the relevant densities and products of densities, then we apply PW-ABC, using both approaches, to four examples: a toy illustrative example of inferring the probability of success in a binomial experiment, a stochastic-differential-equation model, an autoregressive time-series model, and a dynamical predator–prey model. We conclude with a discussion of the strengths and limitations of PW-ABC, and of potential further generalisations.

## Piece-wise ABC (PW-ABC)

Our starting point is to use the Markov property to write the likelihood as

1 $$\begin{aligned} \pi(x \mid \theta) &= \Biggl(\prod_{i=2}^n \pi (x_i \mid x_{i-1}, \ldots,x_1, \theta) \Biggr) \pi(x_1 \mid \theta) \\ &= \Biggl(\prod_{i=2}^n \pi (x_i \mid x_{i-1},\theta) \Biggr) \pi(x_1 \mid \theta). \end{aligned}$$

The likelihood contribution of the first data point *x*_1_ can be included in inference, but this contribution is asymptotically irrelevant as the number of observations, *n*, increases, and we henceforth follow the common practice to ignore the factor *π*(*x*_1_∣*θ*) in (1). Accounting for this, and by using multiple applications of Bayes’ theorem, the posterior density can be written in the following factorised form,

2 $$\begin{aligned} \pi(\theta \mid x) &\propto \pi(x \mid \theta) \pi(\theta) \\ & = \Biggl( \prod_{i=2}^n \frac{\pi( x_i \mid x_{i-1},\theta) \pi(\theta)}{\pi(\theta)} \Biggr) \pi(\theta) \\ &\propto \pi(\theta)^{(2-n)} \Biggl( \prod_{i=2}^n \varphi_i(\theta) \Biggr), \end{aligned}$$

where

$$\begin{aligned} &\varphi_i(\theta) = c_i^{-1} \pi( x_i \mid x_{i-1},\theta) \pi(\theta) \\ &c_i = \int \pi( x_i \mid x_{i-1},\theta) \pi(\theta) \mathrm{d} \theta. \end{aligned}$$

Essentially, in (2) the posterior density, *π*(*θ*∣*x*), of *θ* given the full data *x* has been decomposed into a product involving densities *φ*_*i*_(*θ*), each of which depends only on a pair of data points, {*x*_*i*−1_,*x*_*i*_}.

The key idea now is to use ABC to draw approximate samples from each of the densities *φ*_*i*_(*θ*). Applying Algorithm 2 involves (i) drawing *θ* ^∗^ from *π*(*θ*), (ii) simulating $x_{i}^{*}\mid x_{i-1},\theta^{*}$, and (iii) accepting *θ* ^∗^ if $d (s(x_{i}),s(x_{i}^{*}) ) \leq \varepsilon$. We use $\tilde{\varphi}_{i}(\theta)$ to denote the implied ABC density from which these samples are drawn (with $\tilde{\varphi}_{i}(\theta) = \varphi_{i}(\theta)$ if *s*(⋅)=Identity(⋅) and *ε*=0). By repeating (i)—(iii) we generate samples of, say, *m* draws, $\theta^{*}_{i(1)}, \dots, \theta^{*}_{i(m)}$, from each $\tilde{\varphi}_{i}(\theta)$. Now, suppose that $\hat{\varphi}_{i}(\theta)$ is an estimate, based on the sample $\theta^{*}_{i(1)}, \dots, \theta^{*}_{i(m)}$, of the density $\tilde{\varphi}_{i}(\theta)$ (and hence of the density *φ*_*i*_(*θ*)). Then the posterior density (2) can be estimated by

3 $$ \hat{\pi}(\theta \mid x) = g(\theta) \Big/ \int g(\theta) \mathrm{d} \theta, $$

where

4 $$ g(\theta) = \pi(\theta)^{(2-n)} \Biggl( \prod _{i=2}^n \hat{\varphi}_i(\theta) \Biggr). $$

The steps of PW-ABC are summarised in Algorithm 3.

**Algorithm 3:**
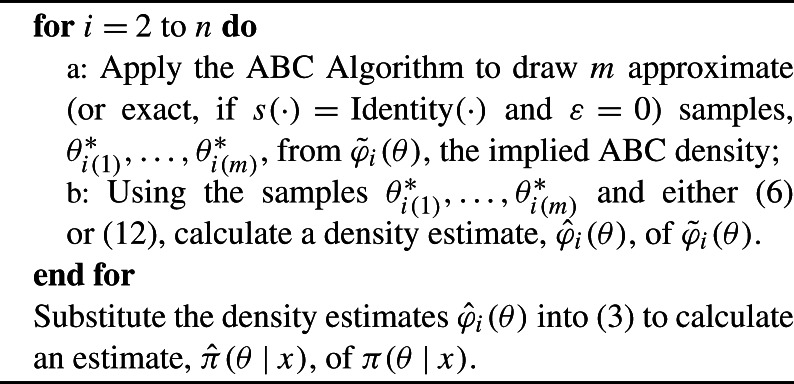
Piece-Wise Approximate Bayesian Computation (PW-ABC)

The rationale of the piecewise approach is to reduce the dimension for ABC, replacing a high-dimensional problem with multiple low-dimensional ones. In standard ABC the summary statistic, *s*(⋅), is the tool used to reduce the dimension, but in PW-ABC, with dimension already reduced by the factorisation in (2), we can take *s*(⋅)=Identity(⋅) and typically use a much smaller *ε*.

The question remains of how to calculate the density estimates, $\hat{\varphi}_{i}(\theta)$. Below we discuss two approaches: (i) using a Gaussian approximation, and (ii) using a kernel density estimate. Henceforth, quantities based on (i) are denoted by superscript g, and those based on (ii) are denoted by superscript k. In both cases we discuss the behaviour of the estimators in the asymptotic regime in which the number of observations, *n*, is kept fixed while the size of each ABC sample increases, *m* →∞.

### Gaussian approximation for $\hat{\varphi}_{i}(\theta)$

Denote the *d*-dimensional multivariate Gaussian density with mean, *μ*, and covariance, *Σ*, by

5 $$\begin{aligned} K(\theta; \mu, \varSigma) =& (2\pi)^{-d/2} (\det \varSigma)^{-1/2} \\ &{}\times\exp \biggl( -\frac{1}{2}{ (\theta-\mu )^T \varSigma^{-1} (\theta-\mu )} \biggr). \end{aligned}$$

A Gaussian approximation for $\hat{\varphi}_{i}(\theta)$ is

6 $$ \hat{\varphi}^\mathrm{g}_i(\theta) = K\bigl(\theta; \bar{ \theta}^*_i, Q_i\bigr), $$

where

$$\begin{aligned} &\bar{\theta}^*_i = \frac{1}{m} \sum _{j=1}^m \theta^*_{i(j)}, \\ &Q_i = \frac{1}{m-1} \sum_{j=1}^m \bigl(\theta^*_{i(j)} - \bar{\theta}^*_i\bigr) \bigl( \theta^*_{i(j)} - \bar{\theta}^*_i\bigr)^T, \end{aligned}$$

are the sample mean and sample covariance of the ABC posterior sample $\theta^{*}_{i(1)}, \dots, \theta^{*}_{i(m)}$. A consequence of using (6) is that the product of the density approximations is also Gaussian (though in general unnormalised):

7 $$ \prod_{i=2}^n \hat{\varphi}^\mathrm{g}_i( \theta) = w \cdot K(\theta; a, B), $$

where

8 $$\begin{aligned} & B = \Biggl( \sum_{i=2}^n Q_i^{-1} \Biggr)^{-1}, \end{aligned}$$

9 $$\begin{aligned} &a = B \Biggl( \sum_{i=2}^n Q_i^{-1} \bar{\theta}^*_i \Biggr), \end{aligned}$$

10 $$\begin{aligned} & w = \det(2 \pi B)^{1/2} \prod_{i=2}^n \det(2 \pi Q_i)^{-1/2} \\ &\phantom{w =\,}\times \prod_{s=2}^n\prod _{t>s}^n\exp \biggl(-\frac{1}{2}{ \bigl(\bar{\theta}^*_s - \bar{\theta}^*_t \bigr)^T R_{st}\bigl(\bar{\theta}^*_s - \bar{ \theta}^*_t\bigr) } \biggr), \end{aligned}$$

11 $$\begin{aligned} & R_{st} = Q_s^{-1} B Q_t^{-1}. \end{aligned}$$

We note the following properties of approximation (6) (see, for example, Mardia et al. 1979). If the densities $\tilde{\varphi}_{i}(\theta)$ from which the $\theta^{*}_{i(1)}, \dots, \theta^{*}_{i(m)}$ are drawn are Gaussian, i.e., $\tilde{\varphi}_{i}(\theta) = K(\theta; \mu_{i}, \varSigma_{i})$, then $\bar{\theta}^{*}_{i}$ and *Q*_*i*_ are unbiased and consistent estimators of *μ*_*i*_ and *Σ*_*i*_, respectively, and hence *a* and *B* are consistent estimators of the true mean and covariance of $\prod \tilde{\varphi}_{i}(\theta)$. More generally, for $\tilde{\varphi}_{i}(\theta)$ which is not necessarily Gaussian, $\bar{\theta}^{*}_{i}$ and *Q*_*i*_ are consistent estimators of the mean and the variance of the Gaussian density, $\hat{\varphi}^{\mathrm{g}}_{i}(\theta)$, which minimises the Kullback–Leibler divergence,

$$\mathrm{KL}\bigl(\tilde{\varphi}_i(\theta)\|\hat{ \varphi}^\mathrm{g}_i(\theta)\bigr) = \int \tilde{ \varphi}_i(\theta) \log \bigl( \tilde{\varphi}_i(\theta) / \hat{\varphi}^\mathrm{g}_i(\theta) \bigr) {\rm d} \theta; $$

i.e., for each *i*, $\hat{\varphi}^{\mathrm{g}}_{i}(\theta)$ is asymptotically the “optimal” Gaussian approximation to $\tilde{\varphi}_{i}(\theta)$. No such relevant optimality holds for the product of densities, however: the (normalised) product of Gaussians, each of which is closest in the KL sense to $\tilde{\varphi}_{i}(\theta)$, is in general not the Gaussian closest to (the normalised version of) $\prod\tilde{\varphi}_{i}(\theta)$; and indeed it may be very substantially different. In other words, as *m*→∞, *a* and *B* do *not* in general minimise

$$\mathrm{KL} \biggl( \biggl\{ \prod \tilde{\varphi}_i( \theta) / \int \Bigl(\prod \tilde{\varphi}_i(\theta) \Bigr) \biggr\} \Big\| K(\theta,a,B) \biggr). $$

### Kernel density estimate for $\hat{\varphi}_{i}(\theta)$

A second method we consider is to estimate each density $\tilde{\varphi}_{i}(\theta)$ using a kernel density estimate (see for instance Silverman 1986 and Wand and Jones 1995). A kernel density estimate based on Gaussian kernel functions (5) is

12 $$\begin{aligned}& \hat{\varphi}_i^\mathrm{k}(\theta) = \frac{1}{m} \sum _{j=1}^m K\bigl(\theta; \theta^*_{i(j)}, H_i \bigr), \end{aligned}$$

where *H*_*i*_ is a bandwidth matrix. We follow the approach of Fukunaga (1972) in choosing the bandwidth matrix such that the shape of the kernel mimics the shape of the sample, in particular by taking *H*_*i*_ to be proportional to the sample covariance matrix, *Q*_*i*_. Using bandwidth matrix

13 $$ H_i = q \cdot m^{-2/(d+4)} Q_i, $$

where *q*>0 is a constant not dependent on *m*, ensures desirable behaviour as the sample size *m*→∞. In particular, in terms of the little-o notation (*a*_*m*_=*o*(*b*_*m*_) as *m*→∞ denotes lim_*m*→∞_|*a*_*m*_/*b*_*m*_|=0) and with *E* denoting expectation, using choice of bandwidth (13), subject to mild regularity conditions on $\tilde{\varphi}_{i}(\theta)$ (Wand and Jones 1995),

14 $$\begin{aligned} & E \bigl\{ \hat{\varphi}^\mathrm{k}_i(\theta) \bigr\} = \tilde{\varphi}_i(\theta) + o(1), \end{aligned}$$

15 $$\begin{aligned} & E \bigl\{ \hat{\varphi}^\mathrm{k}_i(\theta)^2 \bigr\} = \tilde{\varphi}_i(\theta)^2 + o(1). \end{aligned}$$

From (14)–(15), the bias, $\mathrm{b}\{\hat{\varphi}^{\mathrm{k}}_{i}(\theta)\} = E \{ \hat{\varphi}^{\mathrm{k}}_{i}(\theta) \} - \tilde{\varphi}_{i}(\theta)$, the variance, $\mathrm{var}\{\hat{\varphi}^{\mathrm{k}}_{i}(\theta)\} = E \{ \hat{\varphi}^{\mathrm{k}}_{i}(\theta)^{2} \} - E \{\hat{\varphi}^{\mathrm{k}}_{i}(\theta) \}^{2}$, and the mean integrated squared error,

16 $$\begin{aligned} \mathrm{MISE}\bigl\{ \hat{\varphi}^\mathrm{k}_i \bigr\} & = E \int \bigl(\hat{\varphi}^\mathrm{k}_i(\theta) - \tilde{ \varphi}_i(\theta) \bigr)^2 \mathrm{d} \theta, \end{aligned}$$

are all *o*(1). These results generalise routinely to the case of a product of *n* kernel density estimates, that is, in which $\prod \hat{\varphi}^{\mathrm{k}}_{i}(\theta)$ is used as an estimator for $\prod \tilde{\varphi}_{i}(\theta)$. It follows that since the $\theta^{*}_{i(j)}$ are independent for all *i*,*j*, then, using (14)–(15),

$$\begin{aligned} &\mathrm{b} \Bigl\{ \prod \hat{\varphi}^\mathrm{k}_i( \theta) \Bigr\} = \Bigl\{ \prod E \hat{\varphi}^\mathrm{k}_i( \theta) \Bigr\} - \prod \tilde{\varphi}_i(\theta) = o(1), \\ & \operatorname{var} \Bigl\{ \prod \hat{\varphi}^\mathrm{k}_i( \theta) \Bigr\} = \prod E \bigl\{ \hat{\varphi}^\mathrm{k}_i( \theta)^2 \bigr\} - \prod \bigl\{ E \hat{ \varphi}^\mathrm{k}_i(\theta) \bigr\} ^2 = o(1), \\ & \mathrm{MISE} \Bigl\{ \prod \hat{\varphi}^\mathrm{k}_i \Bigr\} = E \int \Bigl( \prod \hat{\varphi}^\mathrm{k}_i( \theta) - \prod \tilde{\varphi}_i(\theta) \Bigr)^2 \mathrm{d} \theta = o(1). \end{aligned}$$

Hence, in the sense defined by the latter equation, the density estimator $\prod \hat{\varphi}^{\mathrm{k}}_{i}(\theta)$ converges to the true density $\prod \tilde{\varphi}_{i}(\theta)$ as *m*→∞.

Regarding the choice of *q* in (13), in certain settings it is possible to determine an optimal value. Suppose that the true density $\tilde{\varphi}_{i}(\theta)$ is Gaussian and let $\hat{\varphi}^{\mathrm{k}}_{i}(\theta)$ in (12) be a kernel density estimate of $\tilde{\varphi}_{i}(\theta)$. Then

17 $$ q = \bigl\{ ( d+2 )/4\bigr\} ^{-2/(d+4)} $$

is optimal in the sense that (13) is then an unbiased and consistent estimator of the bandwidth that minimises the leading term of the large-*m* asymptotic expansion of (16); see Wand and Jones (1995, p. 111). Analogous calculations are rather more involved in the product case, however: even with the assumption that each $\tilde{\varphi}_{i}(\theta)$ is Gaussian, no closed expression for *q* is possible. Hence, in the examples in the following section, Sect. 4, we opted to tune *q* in the heuristic way described by Wand and Jones (1995), starting with a large *q* (ten times that in (17)) then reducing it manually until “random” fluctuations begin to appear in the density estimates.

A consequence of using Gaussian kernel functions (5) in (12) is that the product of the density approximations is then itself a weighted mixture of (*n*−1)^*m*^ Gaussians,

18 $$\begin{aligned} \prod_{i=2}^n \hat{\varphi}^\mathrm{k}_i( \theta) &= m^{(1-n)}\prod_{i=2}^n \sum_{j=1}^m K\bigl(\theta; \theta^*_{i(j)}, H_i \bigr) \\ & = m^{(1-n)} \sum_{j_2, \ldots, j_n}^m \prod _{i=2}^n K\bigl(\theta; \theta^*_{i(j_i)}, H_i \bigr) \\ & = \sum_{j_2,\ldots,j_n}^m w_{j_2,\ldots,j_n} K( \theta; a_{j_2,\ldots,j_n}, B_{j_2,\ldots,j_n}), \end{aligned}$$

where expressions for the covariances $B_{j_{2},\ldots,j_{n}}$, means $a_{j_{2},\ldots,j_{n}}$, and weights $w_{j_{2},\ldots,j_{n}}$, analogous to those in (8)–(10), are given in Appendix 1.

### Estimating the posterior density

Sections 2.1 and 2.2 describe methods for computing the factor $\prod \hat{\varphi}_{i}(\theta)$ in (3). For calculating an estimate of the full posterior, $\hat{\pi}(\theta \mid x)$ in (3), we must multiply $\prod \hat{\varphi}_{i}(\theta)$ by *π*(*θ*)^(2−*n*)^ and normalise. Let us suppose that the prior is Gaussian, *π*(*θ*)=*K*(*θ*;*μ*_pri_,*Σ*_pri_). For the case where we are using the Gaussian approximation, $\hat{\varphi}^{\mathrm{g}}_{i}(\theta)$ from (6), for each $\hat{\varphi}_{i}(\theta)$, then the posterior is

19 $$ \hat{\pi}^\mathrm{g}(\theta \mid x) = K(\theta; \mu_{\mathrm{post}}, \varSigma_{\mathrm{post}}), $$

where

20 $$\begin{aligned} &\varSigma_{\mathrm{post}} = \bigl( (2-n) \varSigma_\mathrm{pri}^{-1} + B^{-1} \bigr)^{-1}, \end{aligned}$$

21 $$\begin{aligned} &\mu_{\mathrm{post}} = \varSigma_{\mathrm{post}} \bigl( (2-n) \varSigma_\mathrm{pri}^{-1} \mu_{\mathrm{pri}} + B^{-1} a \bigr), \end{aligned}$$

and *a* and *B* are as defined in (7).

If instead we use the kernel approximation, $\hat{\varphi}^{\mathrm{k}}_{i}(\theta)$ from (12), for each $\hat{\varphi}_{i}(\theta)$, then the posterior density is

22 $$\begin{aligned} \hat{\pi}^\mathrm{k}(\theta \mid x) =& \sum _{j_2,\ldots,j_n}^m w'_{j_2,\ldots,j_n} K\bigl( \theta; a'_{j_2,\ldots,j_n}, B'_{j_2,\ldots,j_n}\bigr) \\ &{} \Big/ \sum_{j_2,\ldots,j_n}^m w'_{j_2,\ldots,j_n}, \end{aligned}$$

where expressions for $B'_{j_{2},\ldots,j_{n}}$, $a'_{j_{2},\ldots,j_{n}}$ and $w'_{j_{2},\ldots,j_{n}}$ are in Appendix 1.

### An expression for the posterior density

In the preceding sections we considered how to sample from the *φ*_*i*_(*θ*) and then use the samples to estimate the posterior density *π*(*θ*∣*x*). Here we consider in more detail the implied posterior density which is targeted by PW-ABC. For either of PW-ABC and ABC, the posterior can be written as

23 $$ \tilde{\pi}(\theta \mid x) \propto \tilde{\pi}(x \mid \theta) \pi(\theta), $$

where $\tilde{\pi}(x \mid \theta)$ is, respectively, either the implied PW-ABC or ABC approximation to the likelihood. First, we define the function 
24 where argument *z* is of dimension, say, *u*, and either continuous- or discrete-valued in accord with the support of the data; ∥⋅∥_*p*_ is the *L* ^*p*^-norm;  is an indicator function; and *V*, which depends on *u*, *ε*, and *p*, is such that ∫*K*_*ε*,*p*_(*z*)d*z*=1, with this integral interpreted as a sum in the discrete case. For ABC with distance *d*(⋅,⋅) taken to be the *L* ^*p*^-norm of the difference between its arguments, the implied ABC approximation to the likelihood (Wilkinson 2013) is the convolution

25 $$\begin{aligned} &\tilde{\pi}_{\mathrm{ABC}}(x \mid \theta) = \int \pi(y \mid \theta) K_{\varepsilon,p} ( y - x ) {\mathrm{d}} y. \end{aligned}$$

Hence ABC replaces the true likelihood with an approximate version averaged over an *L* ^*p*^-ball of radius *ε* centred on the data vector, *x*. In PW-ABC, we target each *φ*_*i*_(*θ*) by an ABC approximation $\tilde{\varphi}_{i}(\theta) \propto \tilde{\pi}_{\mathrm{ABC}}(x_{i} \mid x_{i-1}, \theta) \pi(\theta)$, with

$$ \tilde{\pi}_{\mathrm{ABC}}(x_i \mid x_{i-1}, \theta) = \int \pi(y_i \mid x_{i-1} , \theta) K_{\varepsilon,p} ( y_i - x_i ) {\mathrm{d}} y_i, $$

and the implied PW-ABC likelihood is the product

26 $$ \tilde{\pi}_{\text{PW-ABC}}(x \mid \theta) = \prod \tilde{ \pi}_{\mathrm{ABC}}(x_i \mid x_{i-1}, \theta). $$

Now, to compare directly the implied ABC and PW-ABC likelihood approximations, we neglect as before the likelihood contribution from the first observation *x*_1_, then denote by *x*′ the vector *x* with *x*_1_ removed (and similar for *y*); hence we can write (25) and (26), respectively, as

27 $$ \int \pi(y_2 \mid x_{1}, \theta) \Biggl[ \prod _{i=3}^n \pi(y_i \mid y_{i-1}, \theta) \Biggr] K_{\varepsilon,p} \bigl( y' - x' \bigr) {\mathrm{d}} y', $$

and

28 $$ \int \pi(y_2 \mid x_{1}, \theta) \Biggl[ \prod _{i=3}^n \pi(y_i \mid x_{i-1}, \theta) \Biggr] K^*_{\varepsilon,p} \bigl( y' - x' \bigr) {\mathrm{d}} y', $$

where

29 $$ K^*_{\varepsilon,p} \bigl( z' \bigr) = \prod _{i=2}^n K_{\varepsilon,p} \bigl( z'_i \bigr). $$

Two differences between ABC and PW-ABC are clear: first, in ABC the conditioning is on the simulated trajectory, whereas in PW-ABC the conditioning is on the data; and second, in PW-ABC the convolution is with respect to a different kernel (29). This implied kernel seems intuitively reasonable; for example, if the *x*_*i*_ are scalar then the convolution in (28) amounts to an averaging over a hypercube of side length 2*ε* centred on *x*′. The difference in the shapes of the regions defined by *K*_*ε*,*p*_(⋅) and $K^{*}_{\varepsilon,p}(\cdot)$ is of secondary importance, however, since PW-ABC enables use of a much smaller *ε* than ABC, so the averaging will be over a much smaller region around *x*′, and the approximate likelihood will typically be much closer to the true.

## Some other considerations

### Practical issues in drawing samples

The independence of the samples $\theta^{*}_{i(j)}$ for all *i*,*j* means that drawing samples for PW-ABC is “embarrassingly parallel”, i.e., the task can be divided easily between multiple cores. For example, one approach is use all available cores simultaneously to sample from $\tilde{\varphi}_{2}(\theta)$ until *m* draws $\theta^{*}_{2(1)}\cdots \theta^{*}_{2(m)}$ are accepted, and then do likewise for $\tilde{\varphi}_{3}(\theta)$, and so on. Another possibility, which requires less coordination between the cores, is to have different cores sampling from different $\tilde{\varphi}_{i}(\theta)$, then reassign cores as appropriate whenever any of the $\tilde{\varphi}_{i}(\theta)$ reaches *m* accepted samples.

Another benefit of the $\theta^{*}_{i(j)}$ being independent is that samples can be reused in the event of deciding retrospectively to perform PW-ABC with a smaller *ε*: the subset of original samples acceptable with the new smaller *ε* can be retained, leaving only the need, for each $\tilde{\varphi}_{i}(\theta)$, to “top-up” the number of samples to *m*. Similarly, samples can obviously be retained given a retrospective decision to use a larger *m*.

### Estimating the marginal likelihood

In some applications, especially when model comparison is of interest, it is useful to compute the marginal likelihood of the data given the model. The marginal likelihood is

30 $$\begin{aligned} \pi(x) & = \int \pi(x \mid \theta) \pi(\theta) \mathrm{d} \theta \end{aligned}$$

31 $$\begin{aligned} & = \Biggl( \prod_{i=2}^n c_i \Biggr) \int \Biggl( \prod_{i=2}^n \varphi_i(\theta) \Biggr) \pi(\theta)^{2-n} \mathrm{d} \theta. \end{aligned}$$

The unknown *c*_*i*_ can be estimated by $\hat{c}_{i} = m/(V M_{i})$, where *M*_*i*_ equals the number of ABC draws necessary in the *i*th interval to achieve *m* acceptances, and *V* is defined in (24); see Appendix 2. For the integral in (31), using the Gaussian approximation (7) leads to

32 $$\begin{aligned} & \int \Biggl( \prod_{i=2}^n \hat{ \varphi}^\mathrm{g}_i(\theta) \Biggr) \pi( \theta)^{2-n} \mathrm{d} \theta \\ & \quad{} = w \cdot (\operatorname{det} B)^{-1/2} \cdot ( \operatorname{det} \varSigma_{\mathrm{post}})^{1/2} \cdot \bigl( \operatorname{det} (2 \pi \varSigma_\mathrm{pri})\bigr)^{(n/2 - 1)} \\ &\qquad{}\times \exp\biggl\{ -\frac{1}{2}(a-\mu_\mathrm{pri})^T \bigl( (2-n)^{-1}\varSigma_\mathrm{pri} + B \bigr)^{-1} \\ &\qquad{}\times(a-\mu_\mathrm{pri}) \biggr\} , \end{aligned}$$

whereas using the kernel approximation (12) gives

33 $$ \int \Biggl( \prod_{i=2}^n \hat{ \varphi}^\mathrm{k}_i(\theta) \Biggr) \pi( \theta)^{2-n} \mathrm{d} \theta = \sum_{j_2,\ldots,j_n}^m w'_{j_2,\ldots,j_n}. $$

### Practical numerical calculations for the kernel approximation

Since expressions (18), (22), (33) for the kernel case involve sums with (*n*−1)^*m*^ terms, these expressions are largely of academic interest and are typically not suitable for practical calculations. For the examples in this paper we used a more direct numerical approach, first writing (4) as

$$g(\theta) = \exp \Biggl( \sum_{i=2}^n h_i(\theta) \Biggr) \pi(\theta), $$

where $h_{i}(\theta) = \log ( \varphi_{i}^{\mathrm{k}}(\theta) / \pi(\theta) )$, and then evaluating *h*_*i*_(*θ*), *π*(*θ*) and hence *g*(*θ*) pointwise on a fine lattice. Performing calculations in this way on the log scale avoids underflow errors and improves numerical stability compared with trying to evaluate (4) directly. As a further check for robustness, we varied the lattice position and resolution to make sure the results were insensitive to the particular choices.

### Sampling from the posterior distribution

In some circumstances it may be desirable to draw samples from the approximate posterior density. In the Gaussian case, drawing from (19) is straightforward. For the kernel case, (22), in principle sampling can be achieved by normalising the weights, randomly choosing a component with probability equal to these normalised weights, then sampling from the selected Gaussian component. But in practice, again, the large number of terms in (22) will typically preclude this approach. Other possibilities include using a Gibbs sampler, or sampling approximately using Gaussian mixtures with fewer components; see Sudderth et al. (2003).

## Examples

In this section we test PW-ABC on synthetic data from four models. The first, as a toy illustrative example, involves inferring from IID data the probability of success in a binomial experiment. Second is the Cox–Ingersoll–Ross model, a stochastic-differential-equation model for which the continuous state variable has known transition density, which we use to investigate PW-ABC with *ε*>0. Third, we consider an integer-valued time series model called INAR(1), a model for which the likelihood is available (albeit awkward to compute) and enables comparison of our approach with a “gold standard” MCMC approach. Finally, we consider a stochastic Lotka–Volterra model, a simple example from a common class of models (which occur, for instance, in modelling stochastic chemical kinetics) in which the likelihood, and therefore many standard methods of inference, are unavailable.

### Binomial model

**Fig. 1:**
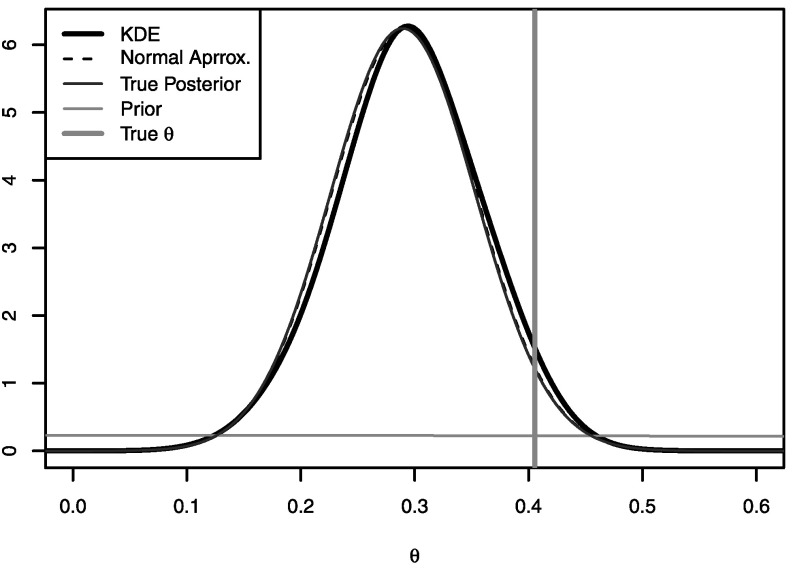
Results for the binomial model in Sect. 4.1. Shown are the true posterior density, *π*(*θ*∣*x*), the posterior density approximations $\hat{\pi}^{\mathrm{g}}(\theta\mid x)$ and $\hat{\pi}^{\mathrm{k}}(\theta\mid x)$, the prior, and the true *θ*

For this toy example we suppose the data is the set *x*={*x*_1_,…,*x*_10_} of *n*=10 observations from the model $X_{i}\sim\operatorname{Binom}(k_{i}=100,p=0.6)$. We work in terms of the transformed parameter $\theta = \operatorname{logit} (p)$, using a prior *π*(*θ*)∼*N*(0,3^2^). For this model the data are IID, so that *π*(*x*_*i*_∣*x*_*i*−1_,*θ*)=*π*(*x*_*i*_∣*θ*). Exact samples from *φ*_*i*_(*θ*) can be obtained by sampling *θ* ^∗^ from the prior, sampling $X_{i}^{*}\sim\operatorname{Binom}(100,\theta^{*})$, and then accepting *θ* ^∗^ if and only if $X_{i}^{*} = x_{i}$. We follow the PW-ABC approach described in Sect. 2, drawing *m*=5000 samples from each *φ*_*i*_(*θ*), using these samples to construct Gaussian $\hat{\varphi}^{\mathrm{g}}_{i}(\theta)$ and kernel density $\hat{\varphi}^{\mathrm{k}}_{i}(\theta)$ approximations, then using these density approximations to construct approximate posterior densities, $\hat{\pi}^{\mathrm{g}}(\theta\mid x)$ and $\hat{\pi}^{\mathrm{k}}(\theta\mid x)$. Figure 1 shows that the approximate posterior densities are very close to the true posterior density for this example. The true log marginal likelihood, log*π*(*x*), computed by direct numerical integration of (30), is −31.39; using approximation $\hat{\varphi}^{\mathrm{g}}_{i}(\theta)$ and (32) gives −31.44; and using approximation $\hat{\varphi}^{\mathrm{k}}_{i}(\theta)$ and numerical integration of the left-hand side of (33) gives −31.48.

### Cox–Ingersoll–Ross Model

The Cox–Ingersoll–Ross (CIR) model (Cox et al. 1985) is a stochastic differential equation (SDE) describing evolution of an interest rate, *X*(*t*). The model is

$$ \mathrm{d}X(t) = a\bigl(b - X(t)\bigr)\mathrm{d}t + \sigma\sqrt{X(t)} \mathrm{d}W(t), $$

where *a*, *b* and *σ* respectively determine the reversion speed, long-run value and volatility, and where *W*(*t*) denotes a standard Brownian motion. The density of *X*(*t*_*i*_)∣*X*(*t*_*j*_), *a*, *b*, *σ* (*t*_*i*_>*t*_*j*_) is a non-central chi-square (Eq. (18), Cox et al. 1985), and hence the likelihood is known in closed form. Since the likelihood is known, (PW-)ABC is unnecessary (indeed, in general for SDEs with unknown likelihoods, approaches that exploit the SDE structure—e.g., the likelihood approximations of Aït-Sahalia (2002), or the Monte Carlo methods developed by Durham and Gallant (2002)—are likely to be better choices for inference than (PW-)ABC); however, we include the CIR model here as a simple example of PW-ABC applied to a problem with a continuous state variable, where non-zero choice of *ε* is necessary, and where the true posterior distribution is available for comparison.

We generated *n*=10 equally spaced observations from a CIR process with parameters (*a*,*b*,*σ*)=(0.5,1,0.15) and *X*(0)=1 on the interval *t*∈[0,4.5]. Treating *a* and *σ* as known, we performed inference on the transformed parameter *θ*=log(*b*) with a Uniform prior on the interval (−5,2). Using *ε*=10^−2^ we drew samples of size *m*=10,000 for each *φ*_*i*_(*θ*), *i*=2,…,10, achieving acceptance rates around 1.5 % on average.

**Fig. 2:**
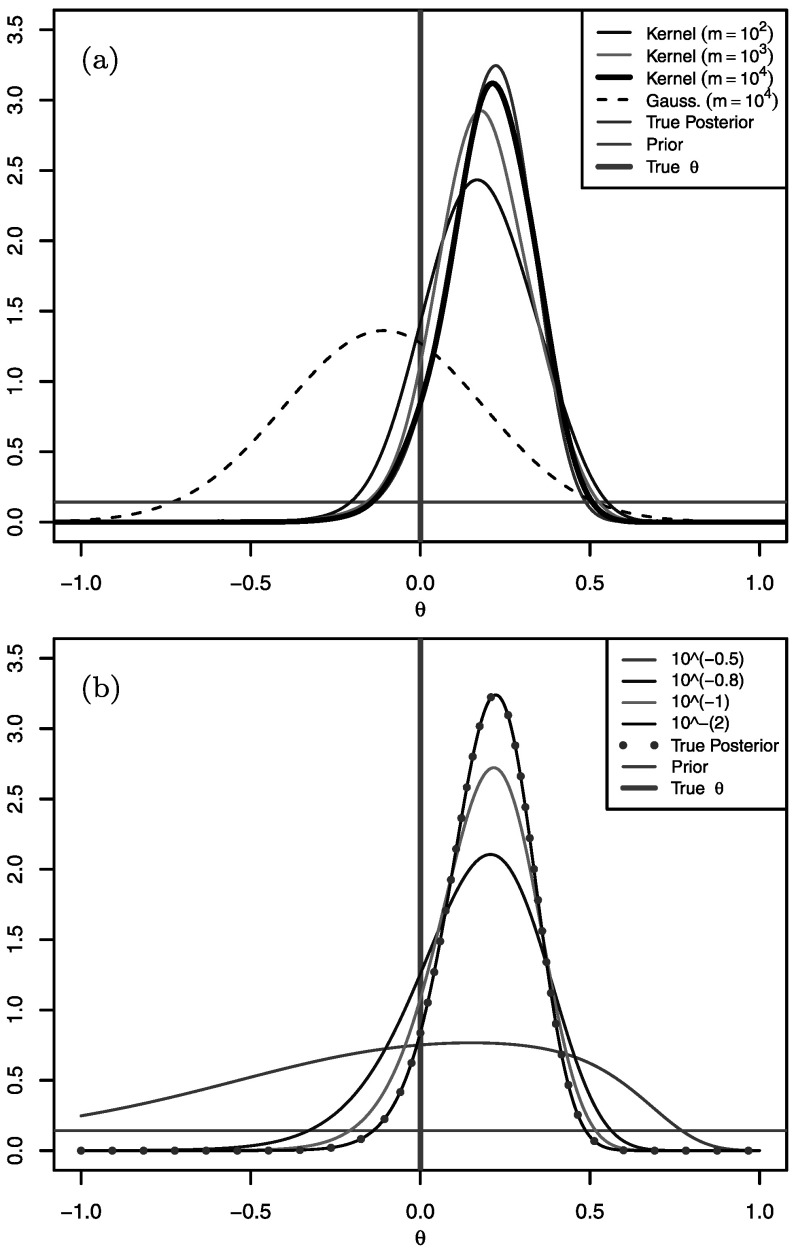
Results for the CIR model of Sect. 4.2. (**a**) shows the true posterior density, *π*(*θ*∣*x*); the PW-ABC posterior density approximations $\hat{\pi}^{\mathrm{g}}(\theta\mid x)$ and $\hat{\pi}^{\mathrm{k}}(\theta\mid x)$ using *ε*=10^−2^, with values of *m* indicated in the legend; the prior; and the true *θ*. (**b**) shows, for various values of *ε*, the true PW-ABC posterior (defined in Sect. 2.4)

Figure 2(a) shows the true posterior density, $\pi(\theta\mid \mathcal{X})$, together with the Gaussian- and kernel-based PW-ABC approximations, $\hat{\pi}^{\mathrm{g}}(\theta\mid x)$ and $\hat{\pi}^{\mathrm{k}}(\theta\mid x)$. The figure shows that for sufficiently large *m* the kernel approximation $\hat{\pi}^{\mathrm{k}}(\theta\mid x)$ agrees very well with the true posterior. The Gaussian approximation $\hat{\pi}^{\mathrm{g}}(\theta\mid x)$, even for large *m*, does badly here, which is due to skewness of the densities *φ*_*i*_(*θ*). Figure 2(b) shows how the posterior density targeted by PW-ABC (see Sect. 2.4) depends on *ε*, and in particular how it converges to the true posterior density as *ε*→0.

For this example, estimates of the log marginal likelihood, $\log \pi(\mathcal{X})$ are as follows: by direct numerical integration of (30), 8.14; using approximation $\hat{\varphi}^{\mathrm{g}}_{i}(\theta)$, 2.78; and by using $\hat{\varphi}^{\mathrm{k}}_{i}(\theta)$ in conjunction with numerical integration of the left-hand side of (33), 7.93.

### An integer-valued autoregressive model

Integer-valued time series arise in contexts such as modelling monthly traffic fatalities (Neal and Subba Rao 2007) or the number of patients in a hospital at a sequence of time points (Moriña et al. 2011). Consider the following integer-valued autoregressive model of order *p*, known as INAR(*p*):

34 $$ X_t = \sum_{i=1}^p \alpha_i \circ X_{t-i} + Z_t, \quad t \in \mathbb{Z}, $$

where *Z*_*t*_ for *t*>1 are independent and identically distributed integer-valued random variables with $E[Z_{t}^{2}] < \infty$, with the *Z*_*t*_ assumed to be independent of the *X*_*t*_. Here we assume *Z*_*t*_∼*Po*(*λ*). Each operator *α*_*i*_∘ denotes binomial thinning defined by

35 $$ \alpha_i \circ W = \left \{ \begin{array}{l@{\quad}l} \operatorname{Binomial}(W,\alpha_i), & W > 0, \\ 0, & W = 0, \end{array} \right . $$

for non-negative integer-valued random variable *W*. The operators *α*_*i*_∘, *i*=1,…*p*, are assumed to be independent.

**Fig. 3:**
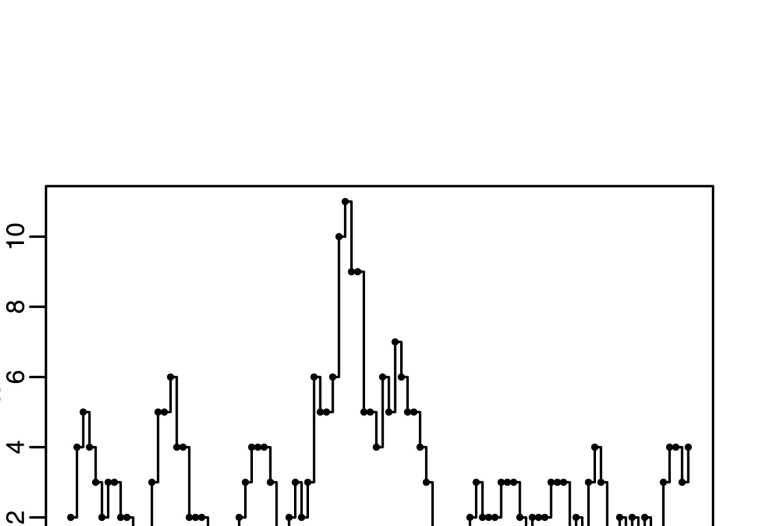
The realisation of an INAR(1) process used in the example of Sect. 4.3, of length *n*=100, generated using *α*=0.7 and *λ*=1.0

We consider the simplest example of this model, INAR(1) (see, for example, Al-Osh and Alzaid 1987), supposing that we have some observed data *x*={*x*_1_,…,*x*_*n*_} from this model and wish to make inference for the parameters (*α*,*λ*). We generated *n*=100 observations from an INAR(1) process using parameters (*α*,*λ*)=(0.7,1) and *X*(0)=10; the realisation is plotted in Fig. 3. Working in terms of the transformed parameter, ${\theta} = (\theta_{1},\theta_{2}) = (\operatorname{logit}(\alpha),\log (\lambda))$, we used a prior of Norm(0,3^2^) for each of *θ*_1_ and *θ*_2_. For the EBC algorithm, the probability of acceptance is around 10^−100^ (as estimated from PW-ABC calculations described below), which is prohibitively small; even the ABC algorithm requires a value of *ε* so large that sequential approaches are needed.

**Fig. 4:**
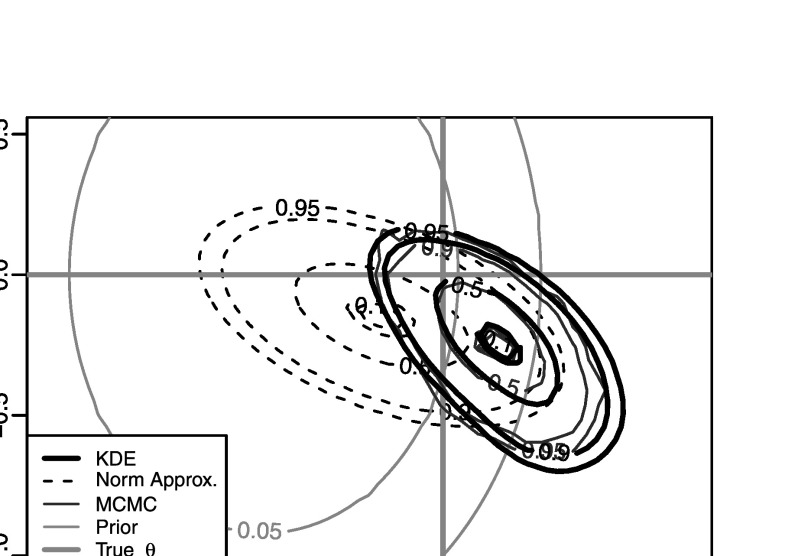
Results for the INAR(1) example of Sect. 4.3. Shown are an MCMC approximation to the posterior density, *π*(*θ*∣*x*), the posterior density approximations $\hat{\pi}^{\mathrm{g}}(\theta\mid x)$ and $\hat{\pi}^{\mathrm{k}}(\theta\mid x)$, the prior, and the true *θ*. The numbers on the contours denote the probability mass that they contain

**Fig. 5:**
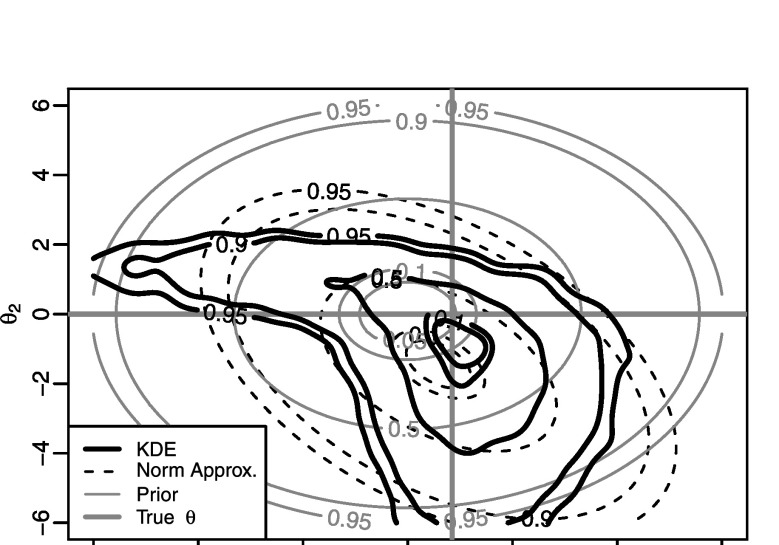
For the INAR(1) example, an example of a factor with a “non-Gaussian” density: here $\hat{\varphi}^{\mathrm{g}}_{50}({\theta})$ and $\hat{\varphi}^{\mathrm{k}}_{50}({\theta})$ are substantially different from each other

Using PW-ABC with *ε*=0 we were able to draw exact samples from *φ*_*i*_(*θ*) for all of the *i*=2,…,100 factors, and achieve acceptance rates of around 9 %, on average. Figure 4 shows an estimate of the posterior density, *π*(*θ*∣*x*) based on a gold-standard MCMC approach, together with Gaussian- and kernel-based PW-ABC approximations, $\hat{\pi}^{\mathrm{g}}(\theta\mid x)$ and $\hat{\pi}^{\mathrm{k}}(\theta\mid x)$, with *m*=10,000 samples for each *φ*_*i*_(*θ*). The figure shows good agreement between the MCMC posterior and the kernel approximation, $\hat{\pi}^{\mathrm{k}}({\theta}\mid x)$, but again somewhat poor agreement with the Gaussian approximation $\hat{\pi}^{\mathrm{g}}({\theta}\mid x)$. The poor performance of $\hat{\pi}^{\mathrm{g}}({\theta}\mid x)$ is caused by some of the densities *φ*_*i*_(*θ*) being substantially different from Gaussian; see Fig. 5 which shows $\hat{\varphi}^{\mathrm{g}}_{50}({\theta})$ and $\hat{\varphi}^{\mathrm{k}}_{50}({\theta})$, for example. Using Gaussian approximations to non-Gaussian *φ*_*i*_(*θ*) appears to have a strong impact on the accuracy of approximation $\hat{\pi}^{\mathrm{g}}({\theta}\mid x)$, even, as in the present case, where the true posterior *π*(*θ*∣*x*), and most of individual *φ*_*i*_(*θ*), are reasonably close to a Gaussian (cf. Fig. 4).

For this example, estimates of the log marginal likelihood, log*π*(*x*), are as follows: by direct numerical integration of (30), −161.1; using approximation $\hat{\varphi}^{\mathrm{g}}_{i}(\theta)$ and (32), −185.7; and by using $\hat{\varphi}^{\mathrm{k}}_{i}(\theta)$ and numerical integration of the left-hand side of (33), −163.2.

We have used *p*=1 for this example so that the likelihood is available, enabling comparison with MCMC and calculation of the true marginal likelihood. However, we stress that PW-ABC can be easily generalised for *p*>1, a case for which the likelihood is essentially intractable and therefore one has to resort to either exact but less direct methods (such the Expectation–Maximization (EM) algorithm or data-augmented MCMC, both of which involve treating the terms *α*_*i*_∘*X*_*t*−*i*_ and *Z*_*t*_ as missing data) or methods of approximate inference, such as conditional least squares which involves minimizing ∑_*t*_(*X*_*t*_−*E*[*X*_*t*_∣*X*_*t*−1_])^2^; see, for example, McKenzie (2003) and references therein.

### Stochastic Lotka–Volterra model

The stochastic Lotka–Volterra (LV) model is a model of predator–prey dynamics and an example of a stochastic discrete-state-space continuous-time Markov process (see, for example, Wilkinson 2011a). Predator–prey dynamics can be thought of in chemical kinetics terms: the predators and prey are two populations of “reactants” subject to three “reactions”, namely prey birth, predation and predator death. Exact simulation of such models is straightforward, e.g., using the algorithm of Gillespie (1977). Inference is simple if the type and precise time of each reaction is observed. However, a more common setting is where the population sizes are only observed at discrete time points. In this case the number of reactions that have taken place is unknown and therefore the likelihood is not available and hence inference is much more difficult. Reversible-jump MCMC has been developed in this context (Boys et al. 2008) but it requires substantial expertise and input from the user to implement. Particle MCMC (pMCMC) methods (Andrieu et al. 2010), which provide an approximation to the likelihood via a Sequential Monte Carlo algorithm within an MCMC algorithm, have recently been proposed for stochastic chemical kinetics models (Golightly and Wilkinson 2011). Although being computationally intensive, such methods can work reliably provided the process is observed with measurement error. The R package smfsb, which accompanies (Wilkinson 2011a), contains a pMCMC implementation designed for stochastic chemical kinetics models, and we use this package to compare results for PW-ABC and pMCMC for the following example.

Let *Y*_1_ and *Y*_2_ denote the number of prey and predators respectively, and suppose *Y*_1_ and *Y*_2_ are subject to the following reactions

36 $$ Y_1 \overset{r_1}{\rightarrow} 2Y_1, \qquad Y_1 + Y_2 \overset{r_2}{\rightarrow} 2Y_2, \qquad Y_2 \overset{r_3}{\rightarrow} \emptyset, $$

which respectively represent prey birth, predation and predator death. We consider the problem of making inference about the rates (*r*_1_,*r*_2_,*r*_3_) based on observations of *Y*_1_ and *Y*_2_ made at fixed intervals.

We generated a realisation from the stochastic LV example of Wilkinson (Wilkinson, p. 208), that is, model (36) using (*r*_1_,*r*_2_,*r*_3_)=(1,0.005,0.6), *Y*_1_(0)=50 and *Y*_2_(0)=100. We performed inference in terms of transformed parameters, *θ*=(*θ*_1_,*θ*_2_,*θ*_3_)=(log*r*_1_,log*r*_2_,log*r*_3_), this time with priors *π*(*θ*_1_)∼Norm(log(0.7),0.5), *π*(*θ*_2_)∼Norm(log(0.005),0.5), and *π*(*θ*_3_)∼Norm(log(0.3),0.5). We again applied PW-ABC using *ε*=0, in other words requiring an exact match between the observed and the simulated observations, to draw samples of size *m*=10,000 for each *φ*_*i*_(*θ*). Unlike the binomial, CIR and INAR examples where drawing posterior samples for the *φ*_*i*_(*θ*), *i*=1,…,*n* assuming *ϵ*=0 took a total of approximately, 1, 2 and 20 minutes respectively on a standard desktop machine, for this example doing so was computationally more demanding. However, since sampling in PW-ABC is embarrassingly parallel (see Sect. 3.1) we were able to draw the required samples in 32 hours on a 48 core machine.

**Fig. 6:**
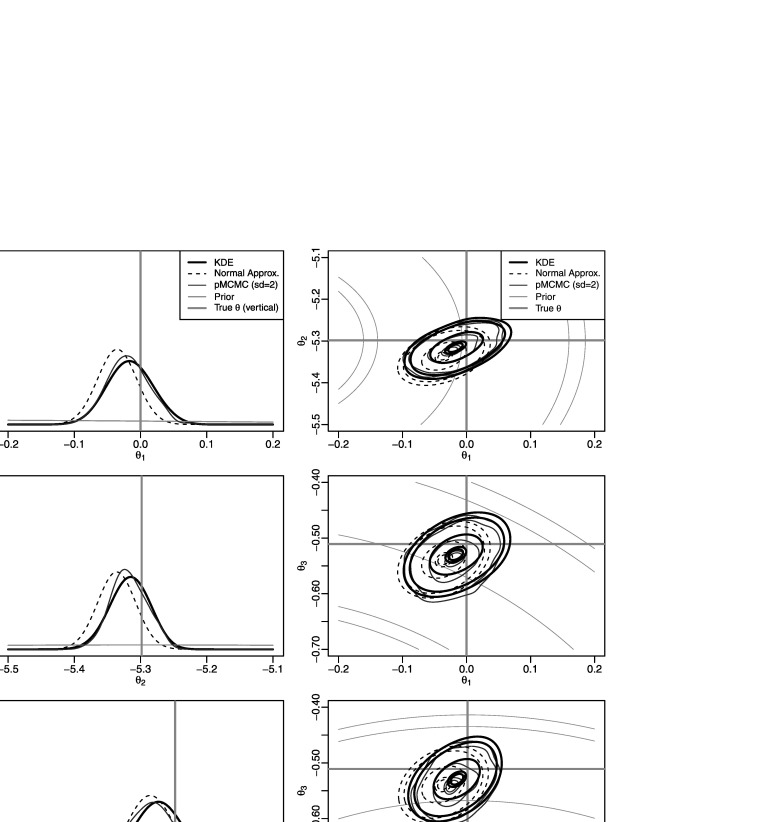
Results for the Lotka–Volterra example of Sect. 4.4, showing univariate and bivariate marginal posterior densities of *θ* based on a posterior sample from a pMCMC algorithm, and from the Gaussian- and kernel-based PW-ABC approximations, $\hat{\pi}^{\mathrm{g}}(\theta\mid x)$ and $\hat{\pi}^{\mathrm{k}}(\theta\mid x)$. For the kernel approximation we used *q*=5 as the smoothing parameter in (13). The contours shown in the bivariate plots are those that contain 5 %, 10 %, 50 %, 90 % and 95 % of probability mass

To obtain pMCMC results we found it necessary to assume an error model for the observations, hence we assumed errors to be IID Gaussian with mean zero and standard deviation equal to 2. Results are displayed in Fig. 6, which shows plots for univariate and pairwise bivariate marginal posterior densities for the pMCMC results, and for the PW-ABC approximations, $\hat{\pi}^{\mathrm{g}}({\theta}\mid x)$ and $\hat{\pi}^{\mathrm{k}}({\theta}\mid x)$. Both of the PW-ABC approximations agree well with each other and with the pMCMC results for this example.

## Conclusion and discussion

PW-ABC works by factorising the posterior density, for which targeting by ABC would entail a careful choice of *s*(⋅) and/or a large tolerance *ε*, into a product involving densities *φ*_*i*_(*θ*), each amenable to using ABC with *s*=Identity(⋅) and small or zero *ε*. Having sampled from each *φ*_*i*_(*θ*) the question then becomes how to estimate *π*(*θ*∣*x*) using these samples. In PW-ABC, we construct density estimates $\hat{\varphi}_{i}(\theta)$ of each *φ*_*i*_(*θ*) then approximate *π*(*θ*∣*x*) as the product of the $\hat{\varphi}_{i}(\theta)$. The approach of taking $\hat{\varphi}_{i}(\theta)$ to be Gaussian, with moments matched to the sample moments, is computationally cheap, and if the prior is also taken to be Gaussian then there is a closed form expression for the Gaussian posterior density and marginal likelihood, making calculations extremely fast. Taking $\hat{\varphi}_{i}(\theta)$ to be Gaussian is perhaps adequate in many applications: performance was strong in two of the four examples we considered. The poor performance in the CIR and INAR examples was due to skewness of at least some of the *φ*_*i*_(*θ*). In the INAR example it is striking to see an effect so strong when the true posterior, and many of the *φ*_*i*_(*θ*), are so close to Gaussian. Unfortunately, increasing the number, *m*, of ABC samples is no remedy to this problem: as *m*→∞, the normalised product of Gaussian densities, itself Gaussian, in general does not converge to the Gaussian density closest in the Kullback–Leibler sense to the target density.

Two referees suggested the possibility of testing, across all of the *φ*_*i*_(*θ*), whether a Gaussian approximation is appropriate. A wide literature exists on testing multivariate normality (see Székely and Rizzo 2005 for a recent contribution, plus many references therein to earlier work) and this seems a promising direction, but further work is needed to devise, and understand the properties of, a procedure based on applying these tests in the multi-testing setting of PW-ABC.

In terms of asymptotic performance, using the kernel approximation, $\hat{\varphi}^{\mathrm{k}}_{i}(\theta)$, for $\hat{\varphi}_{i}(\theta)$ is preferable since, in this case, the estimated posterior density converges to the target as *m*→∞. The kernel approach is computationally more demanding, however, and its practical use is probably limited to problems in which *θ* has small dimension. It also requires a heuristic choice of a scalar smoothing parameter. The larger the value chosen for the smoothing parameter, the more the posterior variance will be inflated; this said, however, in the examples we have considered we have found posterior inference to be fairly robust to the choice. A referee asked for guidance on how to choose *m*. It is difficult to offer general practical advice, because the *m* needed will depend on the dimension of *θ*, and on the number and nature of the *φ*_*i*_(*θ*). The larger the better, of course; one possibility for checking whether *m* is large enough might be to use a resampling approach to confirm that the variance, under resampling, of the $\hat{\pi}^{\mathrm{k}}(\theta\mid x)$ is acceptably small.

Another related practical question is how to choose *ε* if *ε*=0 is not possible. In such a case, as with standard ABC approaches, there is a trade-off between making *m* large and making *ε* small. A reasonable heuristic to investigate the effects of non-zero *ε* would be to perform inference with a chosen *ε* and *m*, and then to keep *m* fixed and reduce *ε* (as discussed in Sect. 3.1, acceptable samples from the run with larger *ε* can be retained), and then check whether there is a marked difference in the posteriors for the different values of *ε*. Figure 2(b) shows for the CIR example, for instance, that there would be little difference between the posteriors for *ε*=10^−2^ and *ε*=10^−3^. Such an approach could be applied iteratively, although for challenging problems—even using PW-ABC—the computational cost to maintain *m* samples as *ε* is decreased may prevent reaching a small enough *ε* that the posterior has “converged” to the true. Such a heuristic could be applied to standard ABC, of course, although PW-ABC has the advantage of enabling much smaller choices of *ε*.

The underlying idea in PW-ABC of replacing a high-dimensional ABC problem with multiple low-dimensional ones is also exploited in some sequential ABC algorithms; for example, Algorithm 4 in Fearnhead and Prangle (2012) (adapted from an algorithm by Wilkinson 2011b) uses ABC to incorporate observations from a Markov model sequentially, the ABC at each step involving a single data point conditional on the previous one, and where the posterior from one step is used as the prior for the next. In comparison with PW-ABC, such sequential algorithms have a potential advantage of progressively focusing computational effort on regions of parameter space with high posterior density, but on the other hand they are prone to problems with particle degeneracy, an issue that does not affect PW-ABC. Another major difference is that for sequential algorithms, samples at each step are dependent, so calculations are not “embarrasingly parallel”, and nor is it so easy to reuse samples in the event of a retrospective decision to use a smaller *ε* or larger *m*; see Sect. 3.1.

A possibility that generalises the Gaussian and kernel approaches in PW-ABC, which we will explore in future work, is to let $\hat{\varphi}_{i}(\theta)$ be a mixture of, say, *u* Gaussians (see Fan et al. 2012 for an example of Gaussian mixtures being used in a related context). This encompasses (6) and (12) as special cases, with *u*=1 and *u*=*m* respectively. For a general mixture model for $\hat{\varphi}_{i}(\theta)$, each of the component Gaussians is parameterised by a scalar weight, a mean vector and a covariance matrix which need to be determined. We would envisage regularising, e.g., by setting each covariance to be equal up to scalar multiplication, perhaps as for (12) taking the covariance proportional to the sample covariance, and then fitting each $\hat{\varphi}_{i}(\theta)$ based on the samples from *φ*_*i*_(*θ*) using, say, an EM algorithm. This approach is a compromise between (6) and (12). It does not share the property of (12) that estimated densities converge to the true densities as *m*→∞, but on the other hand it is computationally much less involved and offers much extra freedom and flexibility over (6), particularly for dealing with multimodal densities. If *u* is taken sufficiently small then it may be feasible to work explicitly with the (*n*−1)^*u*^-term resulting Gaussian mixture, $\prod \hat{\varphi}_{i}(\theta)$, enabling explicit calculations involving the posterior density, such as computing the marginal likelihood, analogous to (32), and direct sampling from the approximate posterior density (see Sect. 3.4).

Several further generalisations of PW-ABC are possible. In (1), each of the *n*−1 factors *π*(*x*_*i*_∣*x*_*i*−1_,*θ*), *i*=2,…,*n* is the likelihood for a single data point conditional on the previous. An alternative possibility is to factorise the likelihood into fewer factors, with each corresponding to a “block” of multiple observations, e.g., $\pi(x_{i+v_{i}}, x_{i+v_{i}-1}, \ldots , x_{i} \mid x_{i-1}, \theta)$ for some choice of *v*_*i*_, and the factorised likelihood becomes a product over the relevant subset of *i*=2,…,*n*. To an extent this potentially reintroduces difficulties that with PW-ABC we sought to avoid, namely lower acceptance rates leading to a possible need to use a summary statistic and non-zero tolerance (and the ensuing ABC error they bring). On the other hand, we might expect, owing to the central limit theorem, that a factor *φ*_*i*_(*θ*) which depends on several data points will be closer to Gaussian than a factor dependent on only a single data point, and hence that (6) and (12) (especially the former) will perform better.

If using larger “blocks” of data in the factorisation makes it necessary to use a non-zero tolerance *ε*>0 (or if *ε*>0 is necessary even when using a single observation per factor) then there are theoretical advantages to using what Fearnhead and Prangle (2012) call “noisy ABC”. In the context of this paper, noisy ABC would involve replacing the summary statistic *s*(⋅) with a random variable *s*′(⋅) which has density uniform on a ball of radius *ε* around *s*(⋅). Using noisy ABC ensures that, under mild regularity conditions, as *n*→∞, the posterior converges to a point mass at the true parameter value; see Sect. 2.2 of Fearnhead and Prangle (2012).

Recently, we have learnt of an interesting paper by Barthelmé and Chopin (2011) who have developed an approach termed *Expectation Propagation-ABC* (EP-ABC) that shares similarities with ours. EP-ABC is an ABC adaptation of the Expectation Propagation approach developed by Minka (2001). EP-ABC uses a factorisation of the posterior (Eq. (1.2) in Barthelmé and Chopin 2011) analogous to our factorisation (2), and it involves a Gaussian approximation to the density of each factor analogous to (6). But then EP-ABC proceeds rather differently: instead of drawing ABC samples for, say, the *i*th factor by sampling from the prior, EP-ABC draws samples from an iteratively updated pseudo-prior. The pseudo-prior is a Gaussian approximation to the component of the posterior that involves all the data *except* those pertaining to the *i*th factor. The use of the pseudo-prior offers a high acceptance rate in the ABC sampling and so EP-ABC can potentially lead to an extremely fast approximation to the full posterior *π*(*θ*∣*x*). A disadvantage is that conditions sufficient for the convergence of EP-ABC (or even the simpler deterministic EP) are not known. Also, as with using PW-ABC with (7), since EP-ABC uses a Gaussian approximation for each factor, it is potentially ill-suited to problems with complicated (e.g. multimodal or otherwise non-Gaussian) likelihoods; convergence of the product density is not assured to any “optimal” approximation to the target posterior. A promising direction for future work will be to investigate adapting the EP-ABC idea of sampling from a pseudo-prior to the ideas in this paper of using kernel (or Gaussian mixture) density estimates for each likelihood factor.

## Appendix 1

Expression for $B_{j_{2},\ldots,j_{n}}$, $a_{j_{2},\ldots,j_{n}}$, and $w_{j_{2},\ldots,j_{n}}$ in (18), analogous to (8)–(10), are as follows:

Expressions for $B'_{j_{2},\ldots,j_{n}}$, $a'_{j_{2},\ldots,j_{n}}$, and $w'_{j_{2},\ldots,j_{n}}$ in (22) are given respectively by the right-hand sides of (20), (21), and (32) with *B* replaced by $B_{j_{2},\ldots,j_{n}}$, *a* replaced by $a_{j_{2},\ldots,j_{n}}$, and *w* replaced by $w_{j_{2},\ldots,j_{n}}$.

## Appendix 2

### Proposition 1

*Let*  *be the indicator function of whether an ABC draw* *θ* ^∗^ *is accepted*. *The acceptance probability is*

$$\mathbb{P}(I=1) = V \tilde{\pi}_\mathrm{ABC}(x) $$

*where* $\tilde{\pi}_{\mathrm{ABC}}(x)$ *is the marginal likelihood of the implied ABC posterior*.

### Proof

Recall from Sect. 2.4 that  and $\tilde{\pi}_{\mathrm{ABC}}(x \mid \theta) = \int \pi(y \mid \theta)\times K_{\varepsilon,p} ( y - x ) {\mathrm{d}} y$ is the implied ABC likelihood approximation. Then

 □

An estimator of $\tilde{\pi}_{\mathrm{ABC}} (x)$ is hence $V^{-1}\hat{\mathbb{P}}(I=1)$, where $\hat{\mathbb{P}}(I=1)$ is the empirical proportion of ABC draws which are accepted.
